# Supplementary figures and images for: Seasonal variation and etiologic inferences of childhood pneumonia and diarrhea mortality in India
Source: eLife. 2019 Aug 27;8:e46202. doi: 10.7554/eLife.46202 (PMC6759316; doi:10.7554/eLife.46202)

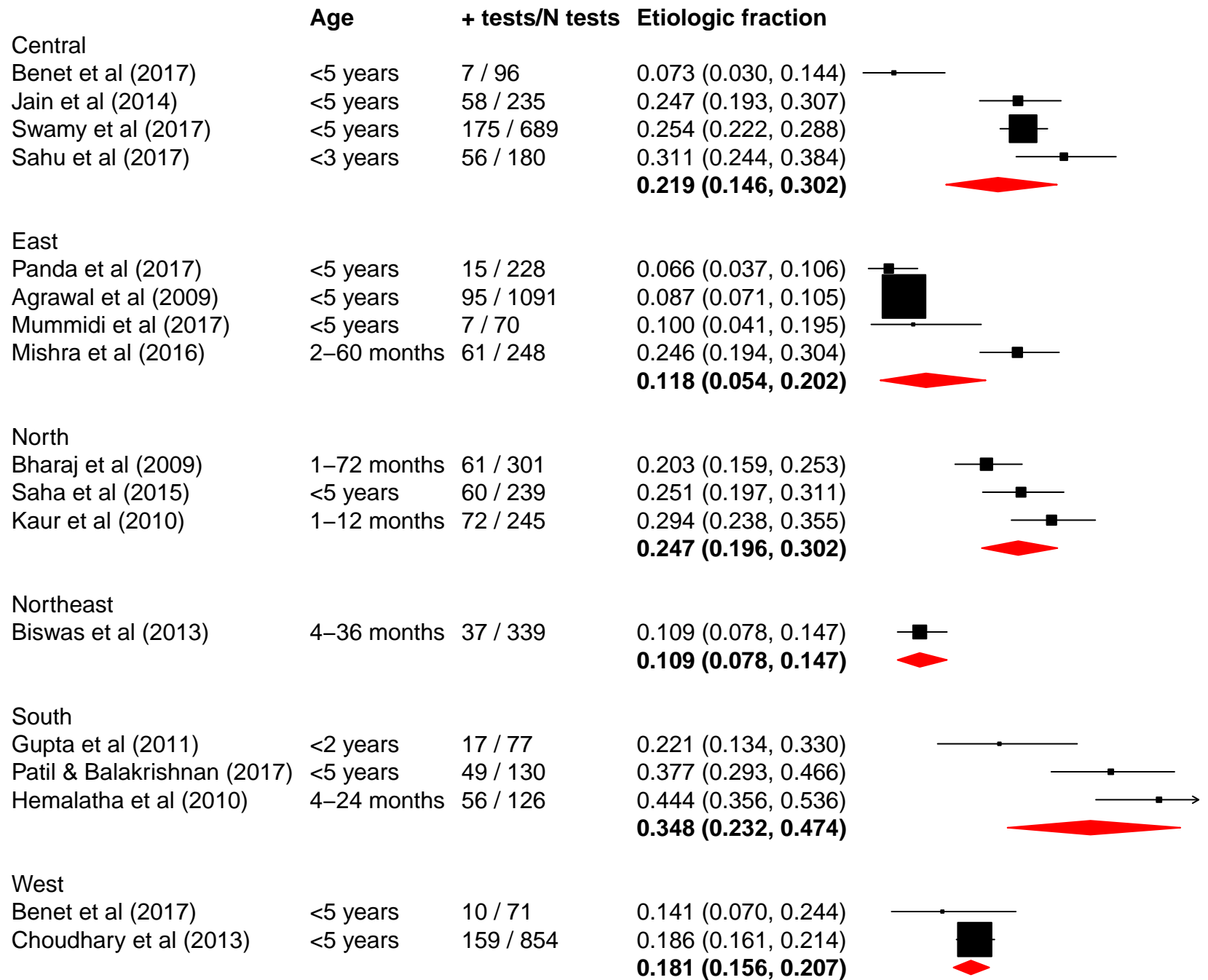

**Total studies = 16**

**Total tests = 5219**

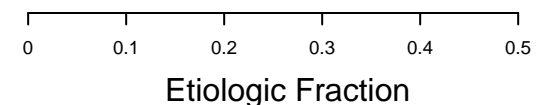

Supplement: Table 4—source data 1. — Studies reporting laboratory testing data were identified through a literature search of Ovid MEDLINE, Scopus, and Google Scholar. All studies described hospitalized children or children seeking care in emergency departments and reported data from 2005 onwards. Studies were meta-analyzed by administrative region using Stata’s metaprop package and visualized in RStudio. We weighted each study using the denominator of total number of laboratory tests in the respective study. [file elife-46202-table4-data1.pdf]

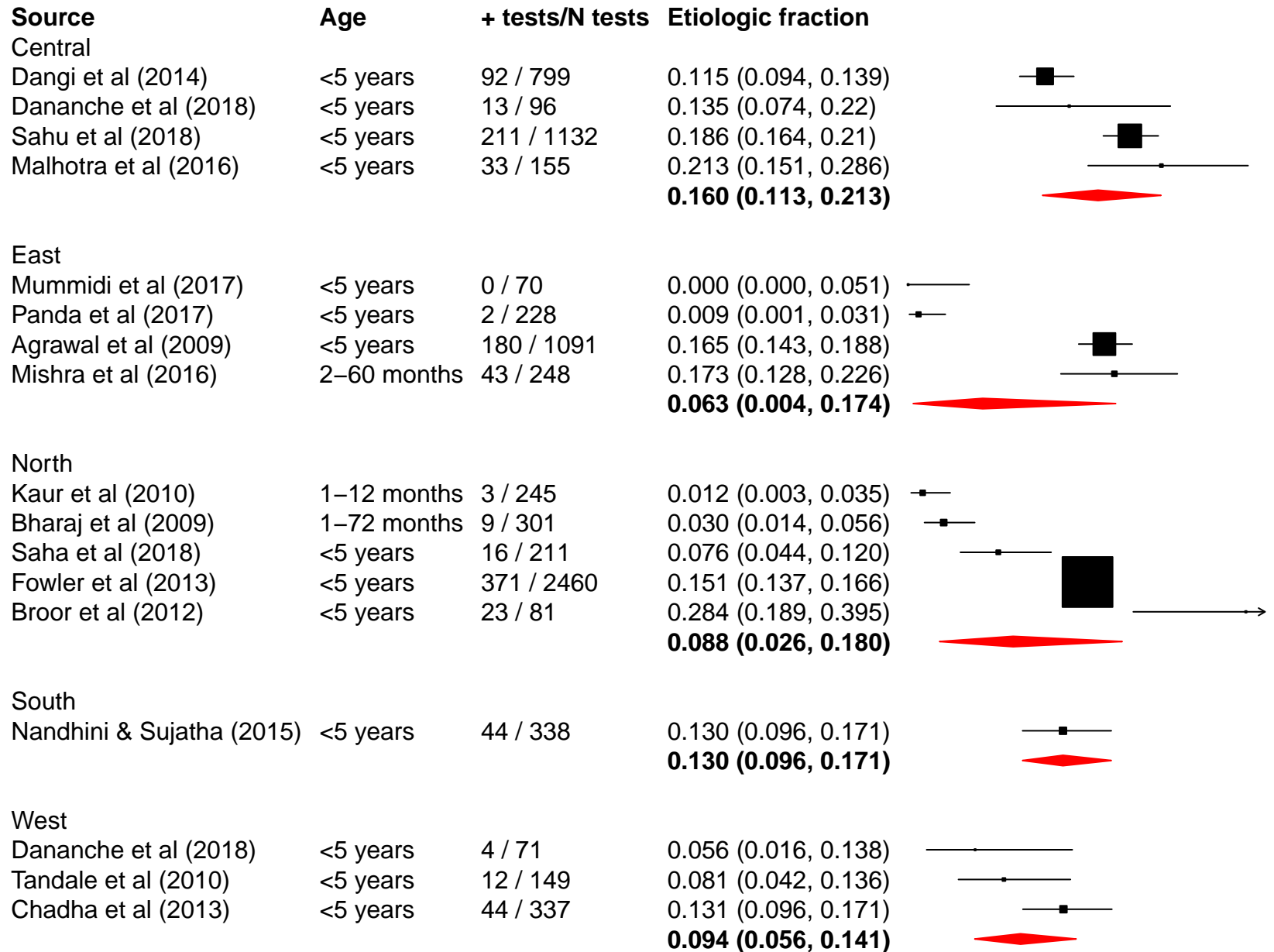

**Total studies = 16**

**Total tests = 8012**

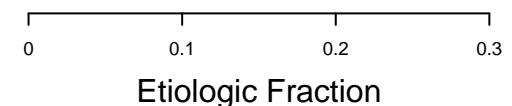

Supplement: Table 4—source data 2. — Studies reporting laboratory testing data were identified through a literature search of Ovid MEDLINE, Scopus, and Google Scholar. All studies described hospitalized children or children seeking care in emergency departments and reported data from 2010 onwards. All studies included describe pandemic and seasonal influenza A and influenza B. Studies were meta-analyzed by administrative region using Stata’s metaprop package and visualized in RStudio. We weighted each study using the denominator of total number of laboratory tests in the respective study. [file elife-46202-table4-data2.pdf]

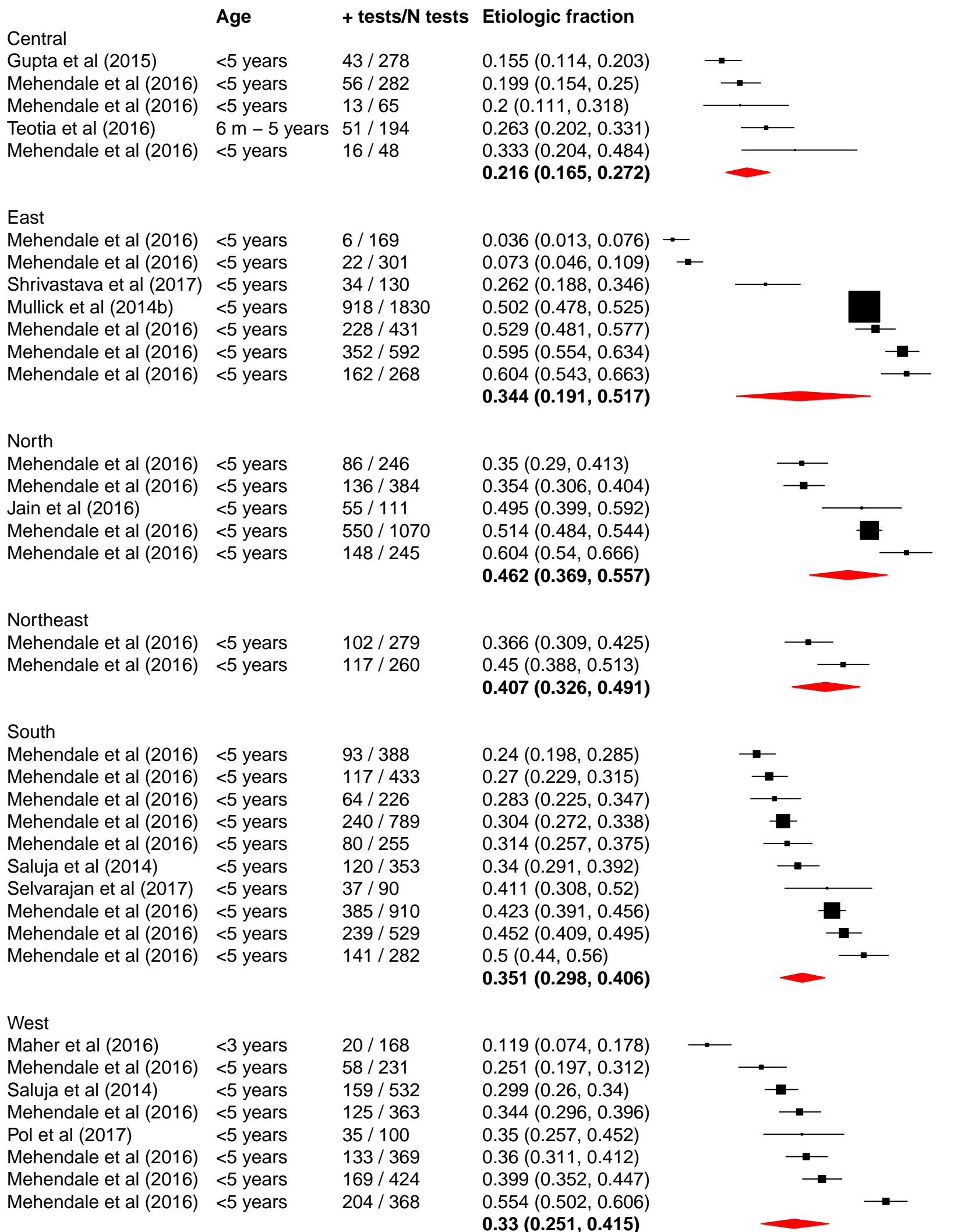

Total studies = 10

Total tests = 13993

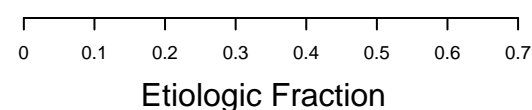

Supplement: Table 4—source data 3. — Studies reporting laboratory testing data were identified through a literature search of Ovid MEDLINE, Scopus, and Google Scholar. All studies described hospitalized children or children seeking care in emergency departments and reported data from 2010 onwards. Studies were meta-analyzed by administrative region using Stata’s metaprop package and visualized in RStudio. We weighted each study using the denominator of total number of laboratory tests in the respective study. [file elife-46202-table4-data3.pdf]
