# Supplementary material for: Seasonal variation and etiologic inferences of childhood pneumonia and diarrhea mortality in India
Source: eLife. 2019 Aug 27;8:e46202. doi: 10.7554/eLife.46202 (PMC6759316; doi:10.7554/eLife.46202)
Supplement: Supplementary file 1. [file elife-46202-supp1.docx]

**References for regional meta-analysis – Respiratory syncytial virus**

Agrawal AS, Sarkar M, Chakrabarti S, Rajendran K, Kaur H, Mishra AC, Chatterjee MK, Naik TN, Chadha MS, Chawla-Sarkar M. 2009. Comparative evaluation of real-time PCR and conventional RT-PCR during a 2 year surveillance for influenza and respiratory syncytial virus among children with acute respiratory infections in Kolkata, India, reveals a distinct seasonality of infection. *J Med Microbiol* **58**:1616–1622. doi:doi:10.1099/jmm.0.011304-0

Benet T, Sanchez Picot V, Messaoudi M, Chou M, Eap T, Wang J, Shen K, Pape JW, Rouzier V, Awasthi S, Pandey N, Bavdekar A, Sanghavi S, Robinson A, Rakoto-Andrianarivelo M, Sylla M, Diallo S, Nymadawa P, Naranbat N, Russomando G, Basualdo W, Komurian-Pradel F, Endtz H, Vanhems P, Paranhos-Baccala G. 2017. Microorganisms Associated With Pneumonia in Children <5 Years of Age in Developing and Emerging Countries: The GABRIEL Pneumonia Multicenter, Prospective, Case-Control Study. *Clin Infect Dis* **65**:604–612. doi:10.1093/cid/cix378

Bharaj P, Sullender WM, Kabra SK, Mani K, Cherian J, Tyagi V, Chahar HS, Kaushik S, Dar L, Broor S. 2009. Respiratory viral infections detected by multiplex PCR among pediatric patients with lower respiratory tract infections seen at an urban hospital in Delhi from 2005 to 2007. *Virol J* **6**:89. doi:10.1186/1743-422X-6-89

Biswas D, Yadav K, Borkakoty B, Mahanta J. 2013. Molecular characterization of human respiratory syncytial virus NA1 and GA5 genotypes detected in Assam in northeast India, 2009-2012: RSV Genotypes in Dibrugarh (Assam), Northeast India. *J Med Virol* **85**:1639–1644. doi:10.1002/jmv.23636

Choudhary ML, Anand SP, Wadhwa BS, Chadha MS. 2013. Genetic variability of human respiratory syncytial virus in Pune, Western India. *Infect Genet Evol* **20**:369–77. doi:10.1016/j.meegid.2013.09.025

Gupta S, Shamsundar R, Shet A, Chawan R, Srinivasa H. 2011. Prevalence of Respiratory Syncytial Virus Infection among Hospitalized Children Presenting with Acute Lower Respiratory Tract Infections. *Indian J Pediatr* **78**:1495–1497. doi:10.1007/s12098-011-0491-0

Hemalatha R, Krishna Swetha G, Seshacharyulu M, Radhakrishna KV. 2010. Respiratory syncitial virus in children with acute respiratory infections. *Indian J Pediatr* **77**:755–758. doi:10.1007/s12098-010-0108-z

Jain B, Singh AK, Dangi T, Agarwal A, Verma AK, Dwivedi M, Singh KP, Jain A. 2014. High prevalence of human metapneumovirus subtype B in cases presenting as severe acute respiratory illness: an experience at tertiary care hospital. *Clin Respir J* **8**:225–33. doi:10.1111/crj.12064

Kaur C, Chohan S, Khare S, Puliyel JM. 2010. Respiratory viruses in acute bronchiolitis in Delhi. *Indian Pediatr* **47**:342–343.

Mishra P, Nayak L, Das RR, Dwibedi B, Singh A. 2016. Viral Agents Causing Acute Respiratory Infections in Children under Five: A Study from Eastern India. *Int J Pediatr* **2016**:1–8. doi:10.1155/2016/7235482

Mummidi PS, Tripathy R, Dwibedi B, Mahapatra A, Baraha S. 2017. Viral aetiology of wheezing in children under five. *Indian J Med Res* **145**:189–193. doi:10.4103/ijmr.IJMR_840_15

Panda S, Mohakud NK, Suar M, Kumar S. 2017. Etiology, seasonality, and clinical characteristics of respiratory viruses in children with respiratory tract infections in Eastern India (Bhubaneswar, Odisha): Epidemiology of Respiratory Viruses in Eastern India. *J Med Virol* **89**:553–558. doi:10.1002/jmv.24661

Patil SL, Balakrishnan A. 2017. Genetic characterization respiratory syncytial virus in Kerala, the southern part of India. *J Med Virol* **89**:2092–2097. doi:10.1002/jmv.24842

Saha S, Pandey BG, Choudekar A, Krishnan A, Gerber SI, Rai SK, Singh P, Chadha M, Lal RB, Broor S. 2015. Evaluation of case definitions for estimation of respiratory syncytial virus associated hospitalizations among children in a rural community of northern India. *J Glob Health* **5**. doi:10.7189/jogh.05.020419

Sahu M, Shukla MK, Barde PV. 2017. Molecular characterization of human respiratory syncytial virus detected from central India. *J Med Virol* **89**:1871–1874. doi:10.1002/jmv.24834

Swamy Ma, Malhotra B, Reddy PJ, Tiwari J, Kumar N, Gupta M. 2017. Trends of respiratory syncytial virus sub-types in children hospitalised at a tertiary care centre in Jaipur during 2012–2014. *Indian J Med Microbiol* **35**:134. doi:10.4103/ijmm.IJMM_15_306

**References for regional meta-analysis – Influenza**

Agrawal AS, Sarkar M, Chakrabarti S, Rajendran K, Kaur H, Mishra AC, Chatterjee MK, Naik TN, Chadha MS, Chawla-Sarkar M. 2009. Comparative evaluation of real-time PCR and conventional RT-PCR during a 2 year surveillance for influenza and respiratory syncytial virus among children with acute respiratory infections in Kolkata, India, reveals a distinct seasonality of infection. *J Med Microbiol* **58**:1616–1622. doi:doi:10.1099/jmm.0.011304-0

Bharaj P, Sullender WM, Kabra SK, Mani K, Cherian J, Tyagi V, Chahar HS, Kaushik S, Dar L, Broor S. 2009. Respiratory viral infections detected by multiplex PCR among pediatric patients with lower respiratory tract infections seen at an urban hospital in Delhi from 2005 to 2007. *Virol J* **6**:89. doi:10.1186/1743-422X-6-89

Broor S, Krishnan A, Roy DS, Dhakad S, Kaushik S, Mir MA, Singh Y, Moen A, Chadha M, Mishra AC, Lal RB. 2012. Dynamic Patterns of Circulating Seasonal and Pandemic A(H1N1)pdm09 Influenza Viruses From 2007–2010 in and around Delhi, India. *PLoS ONE* **7**:e29129. doi:10.1371/journal.pone.0029129

Chadha MS, Hirve S, Dawood FS, Lele P, Deoshatwar A, Sambhudas S, Juvekar S, LaFond KE, Mott JA, Lal RB, Mishra AC. 2013. Burden of Seasonal and Pandemic Influenza-Associated Hospitalization during and after 2009 A(H1N1)pdm09 Pandemic in a Rural Community in India. *PLoS ONE* **8**:e55918. doi:10.1371/journal.pone.0055918

Dananché C, Sánchez Picot V, Bénet T, Messaoudi M, Chou M, Wang J, Pape J-W, Awasthi S, Bavdekar A, Rakoto-Andrianarivelo M, Sylla M, Nymadawa P, Russomando G, Komurian-Pradel F, Endtz H, Paranhos-Baccalà G, Vanhems P, For The Gabriel Network null. 2018. Burden of Influenza in Less Than 5-Year-Old Children Admitted to Hospital with Pneumonia in Developing and Emerging Countries: A Descriptive, Multicenter Study. *Am J Trop Med Hyg* **98**:1805–1810. doi:10.4269/ajtmh.17-0494

Dangi T, Jain B, Singh AK, Singh JV, Kumar R, Dwivedi M, Verma AK, Chadha MS, Jain A. 2014. Molecular characterization of circulating pandemic strains of influenza A virus during 2012 to 2013 in Lucknow (India): Molecular Characterization of Pandemic Strains. *J Med Virol* **86**:2134–2141. doi:10.1002/jmv.23946

Fowler KB, Gupta V, Sullender W, Broor S, Widdowson M-A, Lal RB, Krishnan A. 2013. Incidence of symptomatic A(H1N1)pdm09 influenza during the pandemic and post-pandemic periods in a rural Indian community. *Int J Infect Dis* **17**:e1182–e1185. doi:10.1016/j.ijid.2013.08.005

Kaur C, Chohan S, Khare S, Puliyel JM. 2010. Respiratory viruses in acute bronchiolitis in Delhi. *Indian Pediatr* **47**:342–343.

Malhotra B, Swamy Ma, Janardhan Reddy P, Gupta M. 2016. Viruses causing severe acute respiratory infections (SARI) in children ≤5 years of age at a tertiary care hospital in Rajasthan, India. *Indian J Med Res* **144**:877. doi:10.4103/ijmr.IJMR_22_15

Mishra P, Nayak L, Das RR, Dwibedi B, Singh A. 2016. Viral Agents Causing Acute Respiratory Infections in Children under Five: A Study from Eastern India. *Int J Pediatr* **2016**:1–8. doi:10.1155/2016/7235482

Mummidi PS, Tripathy R, Dwibedi B, Mahapatra A, Baraha S. 2017. Viral aetiology of wheezing in children under five. *Indian J Med Res* **145**:189–193. doi:10.4103/ijmr.IJMR_840_15

Nandhini G, Sujatha S. 2015. Epidemiology of influenza viruses from 2009 to 2013 – A sentinel surveillance report from Union territory of Puducherry, India. *Asian Pac J Trop Med* **8**:718–723. doi:10.1016/j.apjtm.2015.07.028

Panda S, Mohakud NK, Suar M, Kumar S. 2017. Etiology, seasonality, and clinical characteristics of respiratory viruses in children with respiratory tract infections in Eastern India (Bhubaneswar, Odisha): Epidemiology of Respiratory Viruses in Eastern India. *J Med Virol* **89**:553–558. doi:10.1002/jmv.24661

Saha S, Gupta V, Dawood FS, Broor S, Lafond KE, Chadha MS, Rai SK, Krishnan A. 2018. Estimation of community-level influenza-associated illness in a low resource rural setting in India. *PLOS ONE* **13**:e0196495. doi:10.1371/journal.pone.0196495

Sahu M, Singh N, Shukla MK, Potdar VA, Sharma RK, Sahare LK, Ukey MJ, Barde PV. 2018. Molecular and epidemiological analysis of pandemic and post-pandemic influenza A(H1N1)pdm09 virus from central India. *J Med Virol* **90**:447–455. doi:10.1002/jmv.24982

Tandale BV, Pawar SD, Gurav YK, Chadha MS, Koratkar SS, Shelke VN, Mishra AC. 2010. Seroepidemiology of pandemic influenza A (H1N1) 2009 virus infections in Pune, India. *BMC Infect Dis* **10**. doi:10.1186/1471-2334-10-255

**References for regional meta-analysis – Rotavirus**

Gupta S, Singh KP, Jain A, Srivastava S, Kumar V, Singh M. 2015. Aetiology of childhood viral gastroenteritis in Lucknow, north India. *Indian J Med Res* **141**:469–472. doi:10.4103/0971-5916.159298

Jain S, Thakur N, Grover N, Vashistt J, Changotra H. 2016. Prevalence of rotavirus, norovirus and enterovirus in diarrheal diseases in Himachal Pradesh, India. *VirusDisease* **27**:77–83. doi:10.1007/s13337-016-0303-2

Maher G, Pradhan G, Shetty S, Ranshing S, Damle A, Chitambar S. 2016. Rotavirus Infection in Children with Acute Gastroenteritis in Aurangabad, Central Maharashtra. *Indian Pediatr* **53**:631–633.

Mehendale S, Venkatasubramanian S, Girish Kumar CP, Kang G, Gupte MD, Arora R. 2016. Expanded Indian National Rotavirus Surveillance Network in the Context of Rotavirus Vaccine Introduction. *Indian Pediatr* **53**:575–81.

Mullick S, Mandal P, Nayak MK, Ghosh S, De P, Rajendran K, Bhattacharya MK, Mitra U, Ramamurthy T, Kobayashi N, Chawla-Sarkar M. 2014. Hospital based surveillance and genetic characterization of rotavirus strains in children (<5 years) with acute gastroenteritis in Kolkata, India, revealed resurgence of G9 and G2 genotypes during 2011–2013. *Vaccine* **32**:A20–A28. doi:10.1016/j.vaccine.2014.03.018

Pol S, Dedwal A, Ranshing S, Chitambar S, Pednekar S, Bharadwaj R. 2017. Prevalence and characterization of rotaviruses in children hospitalized for diarrheal disease in a tertiary care hospital, Pune. *Indian J Med Microbiol* **35**:33. doi:10.4103/ijmm.IJMM_16_94

Saluja T, Sharma SD, Gupta M, Kundu R, Kar S, Dutta A, Silveira M, Singh JV, Kamath VG, Chaudhary A, Rao JV, Ravi MD, Murthy SRK, Babji S, Prasad R, Gujjula R, Rao R, Dhingra MS. 2014. A multicenter prospective hospital-based surveillance to estimate the burden of rotavirus gastroenteritis in children less than five years of age in India. *Vaccine* **32**:A13–A19. doi:10.1016/j.vaccine.2014.03.030

Selvarajan S, Reju S, Pushpanathan P, Arumugam R, Padmanabhan R, Kothandaramanujam SM, Srikanth P, Kang G. 2017. Molecular characterisation and clinical correlates of rotavirus in children and adults in a tertiary care centre, Chennai, South India. *Indian J Med Microbiol* **35**:221–227. doi:10.4103/ijmm.IJMM_16_51

Shrivastava AK, Kumar S, Mohakud NK, Suar M, Sahu PS. 2017. Multiple etiologies of infectious diarrhea and concurrent infections in a pediatric outpatient-based screening study in Odisha, India. *Gut Pathog* **9**. doi:10.1186/s13099-017-0166-0

Teotia N, Upadhyay A, Agarwal S, Garg A, Shah D. 2016. Rotavirus Diarrhea in Children Presenting to an Urban Hospital in Western Uttar Pradesh, India. *Indian Pediatr* **53**:627–629.
