# Supplementary material for: Seasonal variation and etiologic inferences of childhood pneumonia and diarrhea mortality in India
Source: eLife. 2019 Aug 27;8:e46202. doi: 10.7554/eLife.46202 (PMC6759316; doi:10.7554/eLife.46202)
Supplement: Transparent reporting form [file elife-46202-transrepform.pdf]

1st Floor
24 Hills Road
Cambridge CB2 1JP, UK

P 01223 855340
W elifesciences.org
T @elife

eLife's transparent reporting form

We encourage authors to provide detailed information *within their submission* to facilitate the interpretation and replication of experiments. Authors can upload supporting documentation to indicate the use of appropriate reporting guidelines for health-related research (see EQUATOR Network), life science research (see the BioSharing Information Resource), or the ARRIVE guidelines for reporting work involving animal research. Where applicable, authors should refer to any relevant reporting standards documents in this form.

If you have any questions, please consult our Journal Policies and/or contact us: editorial@elifesciences.org.

Sample-size estimation

- You should state whether an appropriate sample size was computed when the study was being designed
- You should state the statistical method of sample size computation and any required assumptions
- If no explicit power analysis was used, you should describe how you decided what sample (replicate) size (number) to use

Please outline where this information can be found within the submission (e.g., sections or figure legends), or explain why this information doesn't apply to your submission:

Sample size estimation was not conducted for this study as it was not applicable to our study methods, which utilized nationally representative data from India's Sample Registration System between 2005 and 2013. Further details regarding study methods can be found in the 'Materials and Methods' section.

Replicates

- You should report how often each experiment was performed
- You should include a definition of biological versus technical replication
- The data obtained should be provided and sufficient information should be provided to indicate the number of independent biological and/or technical replicates
- If you encountered any outliers, you should describe how these were handled
- Criteria for exclusion/inclusion of data should be clearly stated
- High-throughput sequence data should be uploaded before submission, with a private link for reviewers provided (these are available from both GEO and ArrayExpress)

Please outline where this information can be found within the submission (e.g., sections or figure legends), or explain why this information doesn't apply to your submission:

Pneumonia and diarrhea cases ascertained in this study were identified through dual physician assignment of pneumonia and diarrhea as cause of death. Child deaths where the decedent had a reported history of measles were excluded from all analyses. Child and adult diarrhea deaths assigned as either typhoid or paratyphoid fever were excluded from all analyses except Figure 3 and Figure 3 – figure supplement 1. Inclusion and exclusion criteria for studies included in our meta-analysis are described in the supplementary appendix. Additional details regarding analyses are described in the 'Materials and methods' section.

Statistical reporting

- Statistical analysis methods should be described and justified
- Raw data should be presented in figures whenever informative to do so (typically when N per group is less than 10)
- For each experiment, you should identify the statistical tests used, exact values of N, definitions of center, methods of multiple test correction, and dispersion and precision measures (e.g., mean, median, SD, SEM, confidence intervals; and, for the major substantive results, a measure of effect size (e.g., Pearson's r, Cohen's d)
- Report exact p-values wherever possible alongside the summary statistics and 95% confidence intervals. These should be reported for all key questions and not only when the p-value is less than 0.05.

Please outline where this information can be found within the submission (e.g., sections or figure legends), or explain why this information doesn't apply to your submission:

Statistical analysis methods used in this study are described in the 'Material and methods' section (from lines 302-394) and the supplementary appendix. Further detail regarding specific analyses are provided in figure and table captions, where necessary. Rate ratios and 99% confidence intervals are provided for seasonality figures (i.e. Figure 3, Figure 3 – figure supplement 2, Figure 4, Figure 6, and Figure 8) and in the 'Results' section of the main text.

(For large datasets, or papers with a very large number of statistical tests, you may upload a single table file with tests, Ns, etc., with reference to sections in the manuscript.)

Group allocation

- Indicate how samples were allocated into experimental groups (in the case of clinical studies, please specify allocation to treatment method); if randomization was used, please also state if restricted randomization was applied
- Indicate if masking was used during group allocation, data collection and/or data analysis

Please outline where this information can be found within the submission (e.g., sections or figure legends), or explain why this information doesn't apply to your submission:

Formal group allocation was not conducted for this study given its observational design, using nationally representative death data from 2005 to 2013. Stratified analyses are presented (e.g. by clinical syndrome, age subset, and climate region), and relevant details are provided in the 'Materials and Methods' section.

Additional data files ("source data")

- We encourage you to upload relevant additional data files, such as numerical data that are represented as a graph in a figure, or as a summary table
- Where provided, these should be in the most useful format, and they can be uploaded as "Source data" files linked to a main figure or table
- Include model definition files including the full list of parameters used
- Include code used for data analysis (e.g., R, MatLab)
- Avoid stating that data files are "available upon request"

eLife Sciences Publications, Ltd is a limited liability non-profit non-stock corporation incorporated in the State of Delaware, USA, with company number 5030732, and is registered in the UK with company number FC030576 and branch number BR015634 at the address 1st Floor, 24 Hills Road, Cambridge CB2 1JP | August 2014

Please indicate the figures or tables for which source data files have been provided:

Tables and figures are embedded within the article file submitted through the eLife portal. Source data files have been provided for all seasonality figures; including Figure 3, Figure 3 – figure supplement 1, Figure 3 – figure supplement 2, Figure 4, Figure 4 – figure supplement 1, Figure 6, Figure 6 – figure supplement 1, and Figure 8.
